# Blood–Brain Barrier Dysfunction in a 3D In Vitro Model of Alzheimer's Disease

**DOI:** 10.1002/advs.201900962

**Published:** 2019-08-12

**Authors:** Yoojin Shin, Se Hoon Choi, Eunhee Kim, Enjana Bylykbashi, Jeong Ah Kim, Seok Chung, Doo Yeon Kim, Roger D. Kamm, Rudolph E. Tanzi

**Affiliations:** ^1^ Department of Mechanical Engineering Massachusetts Institute of Technology 500 Technology Square, MIT Building, Room NE47‐321 Cambridge MA 02139 USA; ^2^ Genetics and Aging Research Unit McCance Center for Brain Health Mass General Institute for Neurodegenerative Disease Department of Neurology Massachusetts General Hospital Harvard Medical School Charlestown MA 02129 USA; ^3^ Biomedical Omics Group Korea Basic Science Institute Cheongju 28119 Republic of Korea; ^4^ Department of Bio‐Analytical Science University of Science and Technology Daejeon 34113 Republic of Korea; ^5^ KU‐KIST Graduate School of Converging Science and Technology Korea University Seoul 02841 Republic of Korea; ^6^ School of Mechanical Engineering Korea University Seoul 02841 Republic of Korea; ^7^ Department of Biological Engineering Massachusetts Institute of Technology 500 Technology Square, MIT Building, Room NE47‐321 Cambridge MA 02139 USA; ^8^ Singapore‐MIT Alliance for Research & Technology (SMART) BioSystems and Micromechanics (BioSyM) Singapore 138602 Singapore

**Keywords:** 3D Alzheimer's disease model, Alzheimer's disease, blood–brain barrier

## Abstract

Harmful materials in the blood are prevented from entering the healthy brain by a highly selective blood–brain barrier (BBB), and impairment of barrier function has been associated with a variety of neurological diseases. In Alzheimer's disease (AD), BBB breakdown has been shown to occur even before cognitive decline and brain pathology. To investigate the role of the cerebral vasculature in AD, a physiologically relevant 3D human neural cell culture microfluidic model is developed having a brain endothelial cell monolayer with a BBB‐like phenotype. This model is shown to recapitulate several key aspects of BBB dysfunction observed in AD patients: increased BBB permeability, decreased expression of claudin‐1, claudin‐5, and VE‐cadherin, increased expression of matrix‐metalloproteinase‐2 and reactive oxygen species, and deposition of β‐amyloid (Aβ) peptides at the vascular endothelium. Thus, it provides a well‐controlled platform for investigating BBB function as well as for screening of new drugs that need to pass the BBB to gain access to neural tissues.

## Graphical abstract

**An increase in permeability of the blood–brain barrier (BBB)** has long been considered a key factor in the cause and consequences of Alzheimer's disease (AD). In this study, a physiologically relevant 3D model is developed and used to investigate several key aspects of BBB dysfunction in AD and to provide a standardized platform for screening of new drugs.

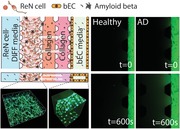

## 1 Introduction

Alzheimer's disease (AD) is the most common form of age‐related dementia, characterized pathologically by deposition of β‐amyloid (Aβ) peptides and development of neurofibrillary tangles in the brain.^1^ In health, the blood–brain barrier (BBB) is highly selective, formed by brain endothelial cells (bECs), preventing harmful materials in the blood from entering the brain. It also regulates transport and clearance of brain Aβ.^2^ Emerging evidence, however, has shown that the BBB is impaired in AD or patients at risk (i.e., those with mild cognitive impairment), and the dysfunctional BBB has been considered a key factor in the cause and consequences of AD.^3^, ^4^, ^5^, ^6^, ^7^, ^8^, ^9^ Therefore, there is tremendous interest in developing cell‐based standardized models to closely mimic BBB alterations observed in AD. Such models would facilitate studies into the function of the BBB in AD and the mechanism(s) by which BBB impairment affects AD pathogenesis. They would also provide a valuable platform to screen for drugs that improve BBB function.

Cell culture models to investigate AD‐associated BBB impairment have previously been developed using cultures of bECs on transwell inserts, incubated with high concentration of synthesized Aβ peptides for short periods. However, these models lack AD neurons and also fail to reproduce the gradual accumulation of soluble Aβ peptides in the extracellular matrix (ECM) and their transport through the local microenvironment.

Microfluidic technology has emerged as a powerful tool for studying new multicellular phenomena with precise control of 3D cellular and noncellular microenvironments, integration of multiple functional steps in one experimental platform, and improved imaging capabilities. We previously developed a 3D human neural cell culture model of AD in which human‐origin neural progenitor cells (NPCs) expressing amyloid precursor protein (APP) or APP/presenilin 1 (PSEN1) with familial AD (FAD) mutations grow to maturity in a 3D culture system (referred as “3D AD” culture).^10^, ^11^ Our 3D AD culture exhibits key events in AD pathogenesis, including extracellular aggregation of Aβ and accumulation of hyperphosphorylated tau. However, this model lacks a BBB, and thus, is limited in its ability to study the role of BBB biology and function in AD.

In the present study, we successfully reproduced the 3D AD culture system with an intact vascular wall in a 3D microfluidic platform to study direct effects of the AD pathological microenvironment on bECs, a major component of the BBB. Our microfluidic AD platform mimics the cerebral–vascular interface by reconstituting a tube‐shaped bEC barrier that has BBB‐like phenotype and integrates into a 3D AD culture system (referred as our “AD model”). Our AD model successfully mimics several vascular alterations observed in AD patients. These include increased BBB permeability with decreased expression of tight junction proteins, claudin‐1 and claudin‐5, and adherens junction (AJ) protein, VE‐cadherin, increased levels of matrix‐metalloproteinase‐2 (MMP‐2) and reactive oxygen species (ROS), and aggregation of Aβ on the abluminal side of the BBB endothelium. To explore the consequences of the impaired BBB in AD pathogenesis, we introduced thrombin, a bloodborne toxic enzyme, within the endothelialized channel. Thrombin was able to pass the leaky BBB and exacerbated neuronal loss in AD, which was prevented by decreasing BBB permeability using pharmacological interventions. We demonstrate that our AD model serves as a well‐controlled platform to understand physiological and pathological mechanisms of BBB dysfunction in AD and can usefully be employed as a standardized therapeutic drug screening platform.

## 2 Results

### 2.1 Development of 3D AD‐BBB in the Microfluidic Platform

We previously reported a unique strategy for recapitulating AD pathology, namely Aβ‐driven neurofibrillary tangles, in a human NPC‐derived 3D Matrigel culture system.^10^, ^11^ Using our 3D system, we found that ReNcell VM human NPCs (ReN cells, Millipore) expressing FAD mutations in the APP gene (ReN‐GA cells) and APP/PSEN1 genes (ReN‐mGAP cells) cause robust extracellular deposition of amyloid plaques and also lead to tauopathy. In the present study, we used the ReN cells derived from the ReN‐GA and ReN‐mGAP, which collectively refer to as ReN‐AD cells. As a control, we used wild‐type ReN (ReN‐WT) cells that do not express FAD mutations.

**Figure 1:**
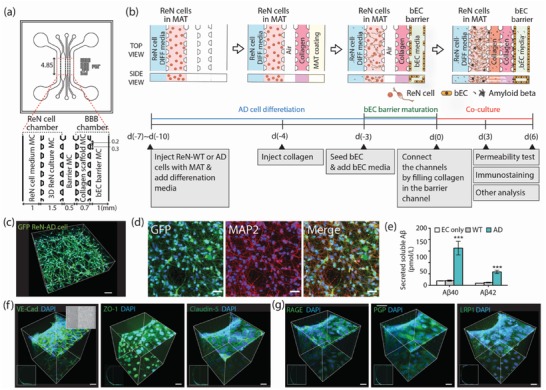
Schematic depiction of a WT/AD model and verification of function of 3D ReN culture and BBB integrity. a) The WT/AD model is composed of ReN‐WT/AD cell and BBB chambers with an intervening barrier microchannel (MC). The ReN cell chamber is composed of a ReN cell medium MC and 3D ReN culture MC, and the BBB chamber is composed of a collagen scaffold MC and bEC barrier MC. Numbers (mm) indicate the dimension of our microfluidic model. b) Sequential experimental protocol for WT/AD model development. The mixture of ReN‐WT or AD cells in Matrigel (MAT) was injected in 3D ReN culture MC, and ReN differentiation medium (DIFF medium) was added in ReN cell media MC. After 3 d, collagen type 1 (4.0 mg mL^−1^) was injected in the collagen scaffold MC, followed by MAT coating (1:50 in serum free media). Next day, bECs (hCMEC/D3) were seeded in the bEC barrier MC, and allowed to form a monolayer on the collagen scaffold and bottom and top of the MC. After monolayer formation, ReN cell and BBB chambers were connected by injecting collagen (2.0 mg mL^−1^) into the barrier MC. c) 3D‐differentiated GFP labeled ReN cells in 3D AD culture MC, and d) the representative image of 2‐week differentiated ReN‐AD cells that are stained with MAP2 (red) and an overlay image. Scale bars: 80 µm in (c) and 40 µm in (d). e) Levels of soluble Aβ40 and Aβ42 detected in media from EC only, ReN‐WT, or ReN‐AD cells in the WT/AD models. f) Expression of junction protein (VE‐Cad) and tight‐junction proteins (ZO‐1 and Claudin‐5), and g) Aβ transporters (RAGE, PgP, and LRP‐1) in the bEC barrier of the AD model. The expression of tight junction proteins and Aβ transporters was confirmed by immunofluorescence staining. Scale bars: 40 µm. Data are mean ± S.E.M. Statistical analysis was by Student's t test.

We developed the AD and WT models by incorporating and coculturing a tube‐shaped bEC barrier with 3D‐differentiated ReN‐AD or ReN‐WT cultures in a 3D microfluidic platform. The microfluidic platform consists of five parallel channels fabricated from polydimethylsiloxane (PDMS) elastomer (**Figure** **1**a). These are grouped into a 3D ReN cell chamber and a BBB chamber, which are positioned on either side of a central “barrier” microchannel (MC) that initially separates the two chambers, but is later filled with hydrogel in order to permit them to interact. The ReN‐AD or WT cell chamber consists of two MCs, one that contains neural cell medium and a second that contains the 3D ReN cell culture. The BBB chamber consists of a collagen scaffold MC and a bEC barrier MC (Figure 1a). All MCs are bounded by an array of posts in order to contain the injected solutions within their respective MCs by the action of surface tension.

To compensate for the different maturation periods and culture conditions required by the ReN cells and the bEC monolayer, and also to prevent the potential influence of ReN‐AD cells on the bEC barrier during monolayer formation, we developed a simple sequential culture method in which the ReN cell and BBB chambers were separated by the barrier MC, an intervening channel initially filled with air, to allow each cell type to mature independently (Figure 1b).

ReN‐AD and ReN‐WT cells were individually mixed with Matrigel (1:1 dilution in Dulbecco's Modified Eagle Medium: Nutrient Mixture F‐12 (DMEM/F12) medium supplemented with B27 and heparin) at a density of 10^7^ cells mL^−1^ and injected into the 3D ReN cell culture MC. After incubation in 5% CO_2_ at 37 °C for 30 min, ReN cell differentiation media was added into the ReN cell media MC. ReN cells were differentiated for 5–7 d before initiating bEC barrier formation.

To form the bEC barrier, a 4 mg mL^−1^ collagen type 1 solution was loaded into the collagen scaffold MC and allowed to gel. On the next day, hCEMC/D3, a well characterized and established brain endothelial cell line,^12^, ^13^ was seeded into the bEC barrier MC and allowed to attach onto the sidewall of the collagen scaffold and bottom and top of the MC. The ReN cell chamber and BBB chamber were maintained separately by keeping the barrier MC filled with air. After the bEC barrier formed a monolayer, the barrier channel was filled with 2 mg mL^−1^ of pH 7.4 collagen type 1 to allow communication between the ReN cells and the bEC monolayer through the ECM via diffusion of soluble factors (Figure 1b; Figure S1, Supporting Information).

Confocal immuno‐histochemical analysis using antibodies against microtubule‐associated protein 2 (MAP2) and glial fibrillary acidic protein (GFAP) showed that most ReN cells differentiated into MAP2‐positive neurons with some GFAP‐positive astrocytes in both the 3D ReN‐AD and ReN‐WT cultures (Figure 1c,d; Figure S2, Supporting Information), confirming successful neuronal differentiation of ReN cells in the 3D ReN culture MC. Enzyme‐linked immunosorbent assay (ELISA) analysis confirmed the secretion of Aβ40 and Aβ42, which were detected in the medium of the ReN cell MC, by ReN‐AD cultures in our AD model (Figure 1e).

Expression and localization of AJs and tight junctions (TJs) of the bEC monolayer were verified with immunofluorescent staining for VE‐cadherin (VE‐cad, an AJ protein), and ZO‐1 and claudin‐5 (both TJ proteins). Our staining analysis confirmed that VE‐cad, ZO‐1, and claudin‐5 were expressed and localized along the cell junction of the bEC monolayer (Figure 1f). We also confirmed the expression of Aβ transporters, receptor for advanced glycation end products (RAGE), P‐glycoprotein, and lipoprotein receptor‐related protein‐1 (LRP1) across the bECs (Figure 1g).

**Figure 2:**
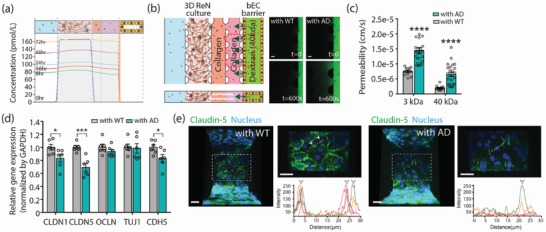
Permeability increase in the AD model. a) The concentration profiles of soluble Aβ secreted from ReN‐AD cells in the AD model. Gray shading indicates the level of accumulated Aβ within ECM and the level of Aβ near the bEC barrier is indicated in the yellow bar. b) Permeability values of the bEC barrier in the WT/AD model measured by introducing bEC medium supplemented with 10 × 10^−6^ m dextran (40 kDa (FITC)) into the bEC barrier MC and monitoring fluorescence every 5 min after flow stabilization. The images were recorded initially (top) and at 600 s (bottom) after adding the dextran. Scale bars: 100 µm. c) Permeability values of the bEC barrier in the WT model and AD model at coculture day 3 (*n* = 13 for (with WT) and *n* = 25 for (with AD) from 3 independent experiments, ****p < 0.0001). d) Relative gene expression levels of tight junction proteins of claudin‐1 and claudin‐5 (CLDN1 & 5), occludin (OCLN), and ZO‐1 (TJU1) and adherens junction protein VE‐cadherin (CDH5) expressed in the bEC barrier in the WT model and AD model (*n* = 6, *p < 0.05, ***p < 0.001, biological replicates  =  3). e) Comparison of tight junction protein expression in the bEC barrier cultured with WT and AD cells in the WT/AD model. The junction protein expression level of cluadin‐5 was visualized by immunofluorescence staining with an antibody against claudin‐5 with DAPI staining for cell nuclei. Graphs show that intensity profiles of distribution of claudin‐5 peaking at the cell junctions (arrows). Each colored line indicates the intensity profile of claudin‐5 expressed by randomly selected cells. Scale bars: 30 µm. Data are mean ± S.E.M. Statistical analysis was by Student's t test.

To further explore the mechanism of Aβ accumulation in the AD model,^14^ a simulation was performed using COMSOL Multiphysics. Secretion of Aβ and gradual accumulation in the ECM in the AD model was simulated as a constant and distributed source of soluble Aβ secreted from ReN‐AD cells at a rate of 1.4 × 10^−12^ mol m^−3^ s^−1^ (5 × 10^−12^ m per hour) (gray box in **Figure** **2**a). Based on the size of soluble Aβ (≈4 kDa), the diffusion coefficient of the bEC barrier was set to 2 × 10^−15^ m^2^ s^−1^, based on the detected amount of soluble Aβ from media in ReN cell media and bEC monolayer MCs (Figure S3, Supporting Information). Daily medium replacement in the bEC barrier MC was also included. All simulation details are presented in the Experimental Section.

### 2.2 BBB Permeability Was Increased in AD Model

We first tested whether our AD models mimic the BBB breakdown observed in AD by directly measuring BBB permeability. Permeability was determined by adding culture medium with 10 × 10^−6^ m fluorescent (FITC)‐labeled or Texas Red‐labeled dextran (*M*_w_: 3 and 40 kDa) in culture media to the bEC barrier MC on days 3 and 6 of coculture, and then imaging the fluorescence every 5 min for more than 30 min. The permeability was calculated by measuring the mean fluorescence intensity in a control volume (CV) defined in the collagen gel immediately after introducing the dextran to the MC and again 10 min later, then applying a permeability equation (Figure 2b; Figure S4, Supporting Information). Our BBB permeability assay indicated that the permeability of the bEC barrier was significantly increased in the AD model (1.4 × 10^−5^ cm s^−1^ for 3 kDa, and 6.45 × 10^−6^ cm s^−1^ for 40 kDa), as compared to the WT model (7.46 × 10^−6^ cm s^−1^ for 3 kDa, and 1.96 × 10^−6^ cm s^−1^ for 40 kDa) (Figure 2c; Figure S5, Supporting Information).

### 2.3 Increased BBB Permeability Was Accompanied by Reduced Expression of Claudin‐1, Claudin‐5, and VE‐cadherin in the bEC of AD Models

We next investigated whether increased BBB permeability was accompanied by disruption of the AJs and TJs of the bEC barrier of the AD models. We screened for AJ and/or TJ gene expression in the bECs of AD models using qRT‐PCR analysis. Since it is difficult to collect sufficient numbers of bECs from the 3D AD/WT models for quantitative reverse transcription polymerase chain reaction (qRT‐PCR) analysis, ReN‐AD or ReN‐WT cells were cultured and differentiated on a 24‐well plate for 5–7 d, and then combined with a bEC monolayer formed on collagen and matrigel‐coated inserts. At coculture day 3, the bECs were collected, and gene expression levels of TJs [CLND1 (claudin‐1), CLND5 (claudin‐5), OCLN (occludin), and TJP1 (ZO‐1)], and the AJ [CDH5 (VE‐cadherin)] were analyzed by qRT‐PCR. Among them, the levels of CLDN1 (claudin‐1), CLDN5 (claudin‐5), CDH5 (VE‐cad) expressions in bECs were significantly decreased when cocultured with ReN‐AD cells compared to those cocultured with ReN‐WT cells (Figure 2d). The levels of OCLN (occludin) and TJP1 (ZO‐1) were not significantly different between WT and AD models. To confirm the reduced expression of tight junction proteins in our AD models, the bEC barrier of AD and WT models was fixed on day 3 of coculture and immunostained with antibodies against claudin‐5 and VE‐cad. Similar to our qRT‐PCR data, expression levels of claudin‐5 and VE‐cad, as viewed from the luminal side of the bEC monolayer formed on the scaffolds in the bEC barrier MC, were reduced at cell–cell junctions in the AD model, compared to the WT model (Figure 2e; Figure S6, Supporting Information). These results suggest that the increase of BBB permeability in our 3D AD model is, at least in part, induced by reduced expression of tight junction and adherens proteins, including claudin‐1 and claudin‐5, and VE‐cad.

### 2.4 Levels of ROS, MMP‐2, and Interferon γ (IFNγ) Were Increased in the AD Models

**Figure 3:**
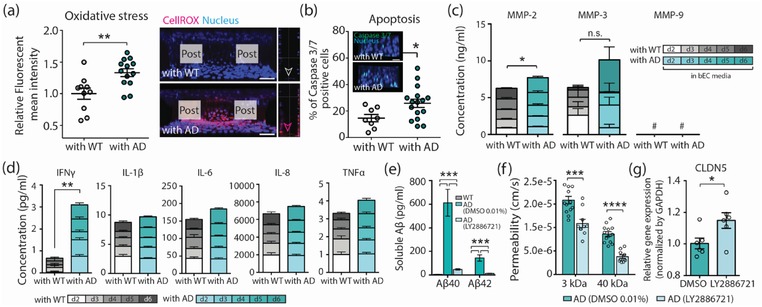
The increased levels of ROS, MMP‐2, and IFNγ in the AD model. a) Relative mean fluorescence intensity of level of intercellular reactive oxygen species (ROS) in the bEC barrier. The level of ROS was confirmed by CellROX Orange. Representative images show that the bEC barrier cocultured with AD cells expressed higher levels of intercellular ROS (indicated with red staining) than those cocultured with WT cells (*n* = 10–13. Biological replicates  =  3, **p < 0.01). Scale bars: 70 µm. b) Apoptosis of bECs were detected by CellEvent Caspase‐3/7 Green Detection Reagent and quantified by counting caspase 3/7 (green) positive cells. Representative images show that the bEC barrier cocultured with AD cells has more caspase 3/7 (green) positive cells. Scale bars: 40 µm (*n* = 8 for WT, *n* = 16 for AD, biological replicates  =  3, *p < 0.05). c) The level of soluble MMPs including MMP‐2, MMP‐3, and MMP‐9 in bEC media in the WT/AD models (*n* = 11–14. Biological repeat = 2, *p < 0.05, n.s., not significant, #, not detected). d) The level of inflammatory cytokines in the WT/AD model (*n* = 8–9, **p < 0.01). e) The levels of Aβ 40 and Aβ 40 secreted by WT cells, AD cells, and AD cells treated with BACE1 inhibitor (LY‐2 886 721) (*n* = 8, ***p < 0.001). f) Permeability of the bEC barrier in the AD model treated with DMSO and BACE 1 inhibitor (LY2886721) (*n* = 16–19, biological replicates =  5, ***p < 0.001, ****p < 0.0001). g) Relative gene expression level of tight junction proteins of claudin‐5 (CLDN5) expressed in the bEC barrier in the AD model with or without LY2886721 treatment (*n* = 3–5, *p = 0.0135). Data are mean ± S.E.M. Statistical analysis was by Student's t test.

Mechanisms by which BBB is impaired in AD should be diverse and complex, though they are currently not clear. Vascular oxidative stress has been suggested to be one of the pathological features that appears in the brains of AD patients.^15^, ^16^, ^17^ Aβ has been shown to produce severe damage to the endothelial lining through deleterious effects on endothelial nitric oxide (NO)^18^, ^19^ and accumulated oxidative stress interferes with increases of endothelial permeability.^20^ Finally, the effect of Aβ has been attributed to enhanced NO catabolism due to an increased production of ROS.^21^, ^22^ Therefore, we measured intracellular ROS levels in the bECs using CellROX orange reagent at coculture day 3. As shown in **Figure** **3**a, we observed that ROS is significantly higher in the bEC barrier of 3D AD models compared to 3D WT models. We also observed an increase in the number of caspase 3/7‐positive apoptotic bECs in the 3D AD model compared to the 3D WT model (Figure 3b).

MMPs are a family of enzymes able to degrade components of the extracellular matrix, which are important for normal BBB function. MMP‐2 has been demonstrated to be a key element in the induction of BBB permeability alterations and disruption of TJs.^23^, ^24^, ^25^ It is secreted by ECs in cultures upon stimulation with Aβ peptides.^26^ AD patients showed higher plasma MMP‐2 levels, although contradicting results have also been reported.^27^, ^28^ We measured the levels of secreted MMP‐2, MMP‐3, and MMP‐9 from day 2 to day 6 of coculture in the bEC media of AD and WT models. Among them, the levels of MMP‐2 were significantly higher in the 3D AD model compared to the 3D WT model (Figure 3c). We did not observe any change in the level of MMP‐3 between the two models, and MMP‐9 was not detectable in either model.

In addition, inflammatory cytokines have been known to contribute to the increase of BBB permeability.^29^, ^30^, ^31^ To determine if the level of inflammatory cytokines increases in our AD model, we measured the levels of IFNγ, interleukin‐1β (IL‐1β), IL‐6, IL‐8, and transforming growth factor α (TNFα), all of which are associated with the increase of BBB permeability, from day 2 to day 6 in the bEC media of our two models. We found that the level of IFNγ detected over 5 d was significantly higher in the media of 3D AD models compared to that of 3D WT models (Figure 3d). Since we did not detect IFNγ in the EC culture medium (data not shown), the detected level of IFNγ is most likely secreted from ReN‐AD cells and had crossed into the bEC barrier. Interestingly, IFNγ has been shown to trigger ROS production^32^, ^33^, ^34^ and also contribute to the increase of BBB permeability.^35^, ^36^

### 2.5 Reducing Aβ Generation Decreased BBB Permeability

To explore whether reducing Aβ generation ameliorates BBB impairment, we treated ReN‐AD cells with BACE1 inhibitor (LY2886721), which prevents the production of Aβ by blocking the β‐secretase enzyme. We added 1 × 10^−6^ m of LY2886721 in the ReN cell differentiation media MC. LY2886721 at this dose dramatically reduced the levels of Aβ40 and Aβ42 secreted from ReN‐AD cells compared to vehicle (0.01% dimethyl sulfoxide (DMSO)) (Figure 3e). We performed a permeability measurement by introducing 3 kDa and 40 kDa FITC dextrans into the bEC barrier MC on day 3 of coculture. The treatment of LY2886721 significantly decreased BBB permeability (Figure 3f) and increased the level of CLDN5 expression in bECs (Figure 3g). These results suggest that Aβ itself and/or Aβ‐driven changes in other molecules play a role in causing BBB impairment.

### 2.6 Deposition of Aβ on the bEC Barrier

**Figure 4:**
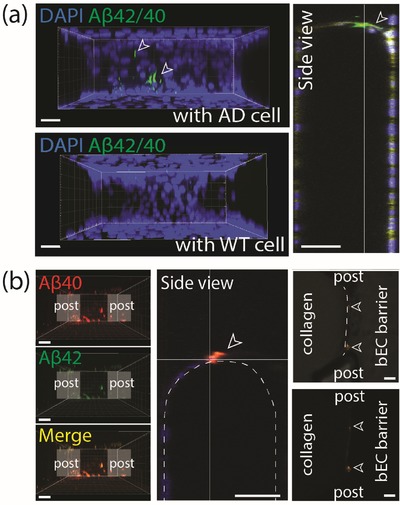
Aβ deposition in the AD model. a) Deposition of Aβ (green) on the BBB monolayer determined by immunofluorescence staining with Aβ 42/40 antibody. White arrows indicate deposited Aβ on the bEC barrier. Scale bars: 30 µm. b) Deposition of flour 555‐labeled Aβ 40 and FAM‐labeled Aβ 42 which were introduced into ReN cell media MC, on the bEC barrier. White arrows indicate deposited Aβ on the bEC barrier. Scale bars: 50 µm.

Aβ deposition is observed in the cerebrovasculature and is usually described as cerebral amyloid angiopathy (CAA). Aβ deposition in the vasculature leads to proinflammatory and cytotoxic events that contribute to the greater BBB permeability in the AD brain. To check whether Aβ species secreted from ReN AD cells are directly deposited onto the bEC barrier in our AD model, we performed immunofluorescent staining with Aβ40 and Aβ42 antibodies at coculture day 6. Aβ deposition was evident in the 3D AD model, but not in 3D WT model (**Figure** **4**a). The total amount of Aβ deposition on the surface of the bEC barrier of our 3D AD model was not extensive, however, possibly due to the short period (7 d) of bEC and ReN cell coculture. To explore which Aβ species, Aβ40 or Aβ42, dominantly deposited on the BBB wall, we introduced Fluor 555‐labeled Aβ40 and FAM‐labeled Aβ42 together in the ReN AD cell media MC and found that both Aβ40 and Aβ42 were codeposited (Figure 4b). These results demonstrate that our AD models successfully mimic the Aβ deposition patterns of CAA around the blood vessels of the brain and might therefore also be useful for studying other functional consequences associated with CAA.

In order to explore whether Aβ can pass the BBB once its permeability is increased, we measured the levels of Aβ40 and Aβ42 in the bEC barrier MC. Although the levels of both were higher in the 3D ReN cell MC of the AD model compared to the WT model (Figure 1e), they were below the detectable range in the bEC barrier MC (Figure S3 of the Supporting Information, left graphs). Thus, it appears that Aβ species deposit and aggregate on the surface of the BBB in our models rather than pass through the disrupted BBB.

### 2.7 The Effects of BBB Disruption on Neuronal Damage

**Figure 5:**
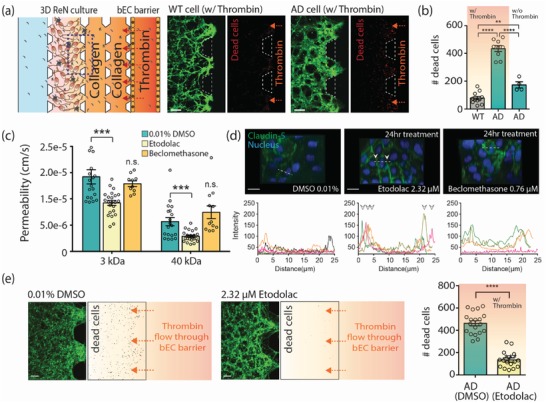
Demonstration of influx of neurotoxins through the impaired BBB and its effects on neural cell damage, and a possible application of drug evaluation for a BBB enhancer. a) Neural cell damage by thrombin inflow through the impaired bEC barrier. Cell death was assayed using ethidium homodimer‐1 after introducing thrombin into the bEC barrier MC at coculture day 3. Dead cells were shown in red. Scale bars: 200 µm. b) The number of dead cells (red) in the ROI (in 0.73 mm^2^) (*n* ≥ 9 for WT/AD with thrombin; *n* ≥ 4 for AD without thrombin, **p < 0.01, ****p < 0.0001). c) Permeability of the bEC barrier cocultured with AD cells with or without drug treatment (*n* ≥ 26 for 0.01 % DMSO and etodoalc, *n* > 5 for beclomethasone, ***p < 0.001, n.s., not significant). d) Comparison of tight junction protein expression in the bEC barrier of the AD model after drug treatment for 24 h. The junction protein expression of claudin‐5 was visualized by immunofluorescence staining with a claudin‐5 antibody and DAPI for cell nuclei. Graphs show the intensity profile of claudin‐5 at the cell junctions. Arrows indicate claudin‐5 expressions at the junctions of cells. Each colored line indicates the intensity profile of claudin‐5 expressed by randomly selected cells. Scale bars: 20 µm. e) Comparison of the amount of dead (damaged) WT/AD neural cells in the 3D ReN culture MC. Cell death was analyzed by counting dead cells (red) in ROI (in 0.73 mm^2^) (*n* ≥ 16, ****p < 0.0001). Scale bars: 100 µm. Data are mean ± S.E.M. Statistical analysis was by Student's t test.

In AD patients, it is possible that circulating neurotoxins enter the brain through the disrupted BBB and exacerbate AD progression. Therefore, enhancing BBB integrity by pharmaceutical drugs might yield beneficial therapeutic effects by blocking the neurotoxins from entering the brain. We tested these hypotheses using our AD models. We first demonstrated that the leakage of neurotoxins through the disrupted BBB can damage neural cells. For this, we introduced thrombin, a serine protease which is directly neurotoxic, that causes proinflammatory effects and whose level is elevated in the brain and cerebral microvasculature in AD,^37^ into the bEC barrier MC on coculture day 3 (**Figure** **5**a). After 3 h of treatment, cell death as determined by ethidium homodimer‐1, increased in the AD models (Figure 5b; Figure S7, Supporting Information). This suggests that thrombin passed through the disrupted BBB to the AD cultures and exacerbated cell death.

Next, we tested whether the disrupted BBB in our system can be restored by pharmacological compounds that enhance BBB integrity. It has been shown that etodolac and beclomethasone have the potential to increase BBB integrity against amyloid toxicity.^38^ We treated the bEC barrier of AD models with either etodolac or beclomethasone at EC50 concentrations of 2.32 and 0.76 × 10^−6^ m, respectively, on coculture day 2. After 24 h, we performed a permeability analysis by introducing 3 and 40 kDa dextran into the bEC barrier MC. Etodolac significantly decreased the permeability of the bEC in the AD model compared to vehicle (0.01% DMSO), while beclomethasone did not show such effects (Figure 5c). Etodolac also significantly increased the expression of claudin‐5 localized between cell–cell junctions of the bEC barrier (Figure 5d). Therefore, etodolac, but not beclomethasone, effectively restored the integrity of the impaired bEC barrier in the AD model.

In order to explore whether increasing BBB integrity pharmacologically has beneficial effects on thrombin‐induced cell death in AD, we treated our AD models with etodolac for 24 h on coculture day 2 and introduced thrombin into the bEC barrier MC on day 3. We observed that the level of dead cells in the 3D AD model treated with etodolac was significantly lower compared to treatment with vehicle (0.01% DMSO) (Figure 5e), leading to the suggestion that drugs that increase BBB integrity might have therapeutic potential for AD and that our 3D AD models can be used to screen those drugs.

## 3 Discussion

Numerous studies have suggested that BBB breakdown initiates and/or contributes to a vicious cycle of AD progression, resulting in neuronal loss and dysfunction.^3^, ^4^, ^5^, ^7^, ^8^, ^9^ Because of the complexity of the in vivo BBB, simplified in vitro models have been developed that mimic barrier impairment observed in AD and offer considerable potential to provide insights into AD pathology and to aid in the development of new drugs for the disease. AD‐associated BBB impairment in a cell culture system has been studied by culturing bECs on transwell inserts and by treating the bECs with high concentrations of synthesized Aβ peptides for short periods.^39^, ^40^, ^41^ However, these classic models lack many features of the human brain in AD and have therefore been of limited value. In the present study, we successfully developed a more physiologically relevant 3D human neural cell culture model of AD with a bEC barrier in a microfluidic platform and verified that it successfully mimics certain aspects of bEC barrier dysfunction, notably the increase in permeability observed in human AD patients.

Our AD model has the following capabilities, which were otherwise difficult to perform with the traditional in vitro BBB transwell models: 1) it provides a 3D microenvironment for cells to quantitatively evaluate bEC barrier permeability; 2) it allows for integration of multiple functional steps in a single experimental platform; 3) it enables enhanced imaging capabilities and economy in reagent and tissue sample size; 4) the developed sequential, compartmentalized culture method allows for the different culture conditions and maturation periods of ReN cells and the bECs; and 5) it realizes a gradual accumulation of Aβ appearing in brains of AD patients by allowing AD cells to continually release Aβ, which gradually accumulates within and diffuse into the ECM. These capabilities enable more physiologically relevant in vitro modeling in our AD model compared with other previous in vitro models.

The paracellular permeability of the bEC barrier was increased in our AD models and it was accompanied by decreased expression of tight junction proteins, claudin‐1 and claudin‐5, and an adherens junction protein, VE‐cadherin. The mechanism(s) of BBB impairment in AD are likely diverse and complex. Aβ might directly increase permeability of the bEC barrier. We found that the levels of oxidative stress, MMP‐2, and IFNγ were increased in our AD model compared to the WT model, all of which have shown to impair BBB function in AD. Interestingly, Aβ did not pass the impaired BBB, but rather deposited on the wall of bECs, mimicking CAA pathology. Future studies will require investigation of whether expression of Aβ transporters is altered on the bECs of our AD model. Reducing Aβ production by the treatment of BACE1 inhibitor, LY‐2 886 721, significantly reduced the permeability of bEC barrier in our model, while it increased the expression level of claudin‐5. These results suggest that Aβ itself and/or Aβ‐driven molecular changes effect BBB dysfunction in AD and that reducing parenchymal Aβ pharmacologically could also reduce BBB leakage in AD.

Using our AD model, we were also able to suggest that increasing BBB integrity pharmacologically might prove to be a useful therapeutic approach for AD treatment by preventing blood‐originated neurotoxins from entering the brain. Accumulation of neurotoxins, such as fibrinogen and thrombin, that enter though impaired BBB from the circulation was observed in AD patients.^42^, ^43^ We first demonstrated that the leakage of neurotoxins through a disrupted BBB exacerbates neuronal death by allowing human thrombin (≈38 kDa) transport across the bEC barrier. These results support the hypothesis that accumulation of neurotoxins in the brains can exacerbate AD pathogenesis.^37^, ^44^ We then showed that enhancing BBB integrity by etodolac significantly decreased the death of ReN‐AD cells induced by thrombin entry. The BBB enhancing effect of beclomethasone was not observed in the AD model, while it significantly enhanced BBB integrity in the transwell model with synthesized Aβ mixture.^38^ This difference in drug effect might be due to the fact that bECs dysfunction in our AD model is affected by not only Aβ peptides but also by other pathological environmental stimuli, such as cytokines from AD cells. If so, it suggests that our model might more realistically replicate in vivo behavior and hence serve as a useful drug screening tool.

We recognize that we do not reconstitute the BBB vasculature entirely. Our model does not contain astrocytes and pericytes both of which play roles in BBB maintenance and function. Rather, in the present study, we have chosen to first study a simpler disease model in order to focus on interactions between the AD cells and bECs, both key components of the BBB. In addition, it has recently been reported that the cerebral vasculature of AD patients has 50% less coverage of pericytes than healthy brains,^45^ and also astrocyte degradation is observed in AD,^46^ which suggests that bECs might be more likely to be directly exposed to environmental toxic stimuli of AD. Therefore, our models will be important in the study of changes in vascular endothelium itself or in its role in AD progression, and how BBB properties are dictated by intrinsic features of bECs in AD pathogenesis.

Recently, attempts have been made to develop more human‐relevant BBB cultures in vitro by employing induced pluripotent stem cells (iPSCs).^47^, ^48^, ^49^, ^50^ However, iPSCs are donor‐specific, and thus highly variable in their behavior, so it would be difficult to develop a standardized BBB model based on iPSCs for drug discovery and development. We chose, therefore, to use well‐characterized and established cell lines, such as hCEMC/D3, in order to develop stable and standardized assays for those purposes with better quality assurance/qualify control (QA/QC).

We believe that our AD model will be a useful standardized tool in the study of BBB biology in AD and also provide a platform for moderate‐throughput drug screening of drugs to inhibit BBB dysfunction or enhance BBB integrity as an adjunct to other AD therapies.

## 4 Experimental Section

*Fabrication of Microfluidic Platform*: The micropatterned master with features 150 µm in height was fabricated by using standard photolithography. SU‐8 100 was coated and patterned on a 4 in. silicon wafer by exposing UV light to the top of a transparent photomask on which the desired patterns are printed. A mixture of PDMS (Sylgard 184, Dow‐Corning, Midland, MI, USA) and its curing agent were mixed to a 10:1 weight ratio, poured onto the patterned master to a 5 mm thickness, degassed to remove air bubbles, and cured in an oven at 80 °C for 1.5 h. The cured PDMS was removed from the wafer and holes were punched through the reservoir patterns to make inlets and outlets for media and hydrogel filling. After sterilization, the devices and glass coverslips were assembled by using oxygen plasma treatment and filled with 60 µL of 1 mg mL^−1^ poly (D‐lysine) solution (PDL, M.W. 70 000–150 000, Sigma‐Aldrich, St. Louis, MO, USA) to strengthen the attachment of hydrogel to the surface. After incubation for 2 h at 37 °C, the PDL solution was removed, and then the devices were rinsed with water and dried at 80 °C overnight.

*Cell Culture*: Human cerebral microvascular endothelial cells (hCMEC/D3, Cedarlane, Ontario, Canada), a cell line derived from human temporal lobe microvessels, were cultured to 90% confluence on a 150 µg mL^−1^ collagen type 1 coated flask in EndoGRO‐MV Complete Culture Media Kit (Millipore, Temecula, CA, USA) supplemented with 1 ng mL^−1^ bFGF (R&D System, Inc., Minneapolis, MN, USA) and 1% penicillin‐streptomycin. ReN‐WT and ReN‐AD cells were cultured on a Matrigel coated flask in proliferation media; DMEM/F12 (Gibco/Life Technologies, Gaithersburg, MD, USA) supplemented with B27 (Life Technologies, Carlsbad, CA, USA), heparin (2 mg mL^−1^ stock, STEMCELL Technologies, Carlsbad, CA, USA), 20 ng mL^−1^ epidermal growth factor (EGF), and 20 ng mL^−1^ fibroblast growth factor 2 (FGF‐2).

*3D Cell Culture in the Microfluidic Platform*: ReN‐WT and ReN‐AD cells in a 25T confluent flask were washed with D‐PBS and treated with Accutase (Life Technologies, Carlsbad, CA, USA) in a 37 °C CO_2_ incubator for 3–5 min for dissociation of cells. The prewarmed differentiation medium was added to the flask, and the cell suspensions were transferred into 15 mL tubes and centrifuged at 2000 g for 3 min. The cells were resuspended at a density of 2 × 10^7^ cells mL^−1^ in the differentiation medium and placed on ice. ReN cell suspensions were mixed with cold Matrigel at the volume ratio of 1:1 (final density: 1 × 10^7^ cells mL^−1^), introduced into the 3D AD culture MC, and placed in a 37 °C CO_2_ incubator for 30 min. After 30 min, the prewarmed differentiation medium was injected into the ReN cell media MC. After 5–7 d, 4 mg mL^−1^ collagen type 1 solution, which was prepared by diluting high concentration collagen type 1 (rat tail, cat. No. 354 249, Corning, NY, USA) in 10× PBS and sterilized deionized water and titrated to pH 7 with 0.5 N NaOH, was injected into the collagen scaffold MC and incubated at 37 °C and 5% CO_2_ for 30 min. After 1 d, bEC barrier MC was coated with growth factor reduced Matrigel (1:50 diluted in serum free media, Corning, NY, USA) for 40 min and hCMEC/D3 cells were seeded at a density of 2 × 10^6^ cells mL^−1^ on the sidewall of the collagen scaffold and top and bottom surfaces of the bEC barrier MC. hCMEC/D3 cells were allowed to form a tight monolayer along the MC for 2–3 d in hCMEC/D3 growth medium. After bEC barrier formation, ReN cell chamber and BBB chamber were connected and allowed to communicate by filling 2 mg mL^−1^ of pH 7 collagen gel (rat tail, cat. No. 354 249, Corning, NY, USA) into the barrier MC. After connection, hCMEC/D3 cells were cultured in EndoGRO‐MV Complete Culture Media Kit (Millipore, Temecula, CA, USA) supplemented with 0.5% fetal bovine serum (FBS), 1 ng mL^−1^ bFGF (R&D System, Inc., Minneapolis, MN, USA), and 1% penicillin–streptomycin.

*Permeability Measurement*: The permeability was determined by adding hCMEC/D3 culture medium with 10 × 10^−6^ m FITC‐labeled dextran (*M*_w_: 40 kDa) into the bEC barrier MC 3 and 6 d after connecting the ReN cell chamber and the BBB chamber. Fluorescent images were recorded every 5 min for 2 h with a confocal microscope. The permeability was estimated by measuring the mean fluorescence intensity in a CV defined in the collagen gel at time 0 and time 600 s and then applying the equation

(1) $$P=\frac{V_{g}}{A_{v}}\left(1/\Delta I_{m}\right)\frac{d}{dt}\bar{I_{g}}$$

where *V*_g_ is gel volume in the CV, *A*_v_ is vessel surface area in CV, $\bar{I_{g}}$ is fluorescence intensity in gel, *I*_m_ is fluorescence intensity at the monolayer (*I*_m_ = *I*_lumen_ − *I*_gel_) at time 0, and $\frac{d}{dt}\bar{I_{g}}$ is the rate of increase in intensity as the dextran diffuses out of the bEC barrier MC into the gel (Figure S4, Supporting Information).

*Reconstitution and Treatment of Aβ40 and Aβ42*: Vacuum‐dried synthetic monomeric Aβ40 and Aβ42, which were obtained from Dr. Robert Moir (Massachusetts General Hospital), were reconstituted by adding 200 µL of 20 × 10^−3^ m NaOH, mixing gently multiple times then allowing 30 s for settling. After several mixing steps, the reconstituted Aβ40 and Aβ42 solutions were centrifuged at 10 000 rpm for 1 min and diluted to the desired concentration with sterilized water. Matrigel (diluted in ReN cell differentiation medium at the volume ratio 1:1) was injected into the 3D AD culture MC, collagen scaffold was then introduced to the collagen scaffold MC, and after Matrigel coating hCMEC/D3 was seeded and the monolayer formed. After monolayer formation, the Aβ40 and Aβ42 solutions were introduced into the ReN cell media MC with ReN cell differentiation medium, after which the ReN cell and BBB chambers were connected.

*Fluorescent‐Tagged Aβ40 and Aβ42 Treatment for CAA Model*: Synthetic human HiLyte Fluor 555‐labeled Aβ40 and FAM‐labeled Aβ42 (Anaspec, San Jose, CA, USA) were reconstituted in 1% NH_4_OH and diluted to the desired concentration of 500 ng mL^−1^ with ReN cell differentiation media. The reconstituted synthetic human HiLyte Fluor 555‐labeled Aβ40 and FAM‐labeled Aβ42 were added into the ReN cell media MC and refreshed daily for 6 d.

*Immunofluorescent Staining and Image Acquisition*: Cells cultured in the microfluidic system were fixed by incubating with 4% (w/v) paraformaldehyde in PBS for MAP2; GFAP; VE‐cad; ZO‐1; RAGE; pgp; LRP1; Aβ40/42 antibodies or methanol for claudin‐5 antibody for 15 min, and permeabilized with 1% Trion‐x100 (Sigma‐Aldrich) in PBS for 5 min. After blocking overnight at 4 °C, primary antibodies including anti‐MAP2 (1:50, Cell Signaling), anti‐GFAP (1:100, Abcam); anti‐VE‐cadherin (1:100, Enzo Lifesciences); anti‐ZO‐1 (10 µg mL^−1^, Invitrogen); anti‐claudin‐5 (5 µg mL^−1^, Abcam); anti‐RAGE (0.6 mg mL^−1^; Abcam); anti‐pgp, (1:500, PBS, Sigma‐Aldrich); anti‐LRP1(1:100, Abcam); and anti‐Aβ40/42 (1:100, Cell Signaling) were added and incubated overnight at 4 °C. After washing the MCs with PBS at least two times, secondary antibodies including Alex Fluor‐488‐conjugated goat‐rabbit and goat‐mouse IgG (1:200, Molecular Probes); Alex‐647‐conjugated goat‐rabbit and goat‐mouse IgG were introduced to the channels and incubated at RT for 2 h, followed by 6‐diamidino‐2‐phenylindole (DAPI, Sigma‐Aldrich) for cell nuclei staining.

*Intracellular ROS Measurement*: Intracellular ROX production was measured by CellROX Orange Reagent (Life Technologies, City, MD, USA) according to manufacturer's protocol. At coculture day 3, HCMEC/D3 cells in the bEC barrier MC were incubated with 5 × 10^−6^ m of CellROX Orange Reagent (Invitrogen, Carlsbad, CA, USA) for 30 min at 37 °C in the dark, followed by triple washing with prewarmed PBS. Then, cells were examined with a confocal laser scanning microscope (FMV‐1000, Olympus, Japan) at an excitation/emission wave‐length of 545/565 nm.

*Cell Apoptosis Assay*: On coculture day 3, HCMEC/D3 cells in the bEC barrier MC were incubated with 5 × 10^−6^ m of CellEvent Caspase‐3/7 Green Detection Reagent (Invitrogen, Carlsbad, CA, USA) with Hoechst for 30 min at 37 °C in the dark and washed twice with prewarmed PBS. Cells were imaged with a confocal laser scanning microscope (FMV‐1000, Olympus, Japan) at excitation/emission wave‐length of 502/530 nm for CellEvent Caspase‐3/7 Green Detection Reagent and 361⁄497 nm for Hoechst.

*Real‐Time Quantitative Reverse Transcription PCR*: Total RNA was isolated and purified using the RNeasy Mini kit (Qiagen, Chatsworth, CA, USA) according to manufacturer's instructions. The concentration of total RNA was measured using a Nanodrop 1000 Spectrophotometer. cDNA was synthesized using High‐Capacity RNA‐to‐cDNA Kit (Applied Biosystems, Foster City, CA, USA) according to manufacturer's protocol. Quantitative Real‐time RT‐PCR was performed on a 7900HT Fast Real‐Time PCR System (Applied Biosystems, Foster City, CA, USA) using TaqMan Fast Advanced Master Mix (Applied Biosystems, Foster City, CA, USA). GAPDH was used as a housekeeping gene. Expressions of the following genes in hCMEC/D3 cells were quantified using TaqMan Gene Expression Assays (Applied Biosystems, Foster City, CA, USA): GAPDH (Hs03929097_g1), CLDN1 (Hs00221623_m1), CLDN5(Hs00533949_s1), OCLN(Hs00170162_m1), ZO‐1(Hs01551861_m1), CDH5 (Hs00901463_m1).

*Computational Simulation of Concentration Profile of Aβ in the AD‐BBB Model*: Concentration profiles of Aβ within the microfluidic system were simulated using COMSOL Multiphysics 5.2. The changes of the concentration profiles over time were estimated by Fick's second law

(2) $$\frac{\partial C}{\partial t}=\;D\frac{\partial^{2}C}{\partial x^{2}}$$

where *C* is concentration and *D* is the diffusion coefficient. Aβ size was taken as 4 kDa, and the diffusion coefficient of Aβ in media, Matrigel, 2 mg mL^−1^ collagen, and 5 mg mL^−1^ collagen were taken from the literature to be 1.8 × 10^−10^, 1.24 × 10^−10^, 0.7 × 10^−10^, and 0.62 × 10^−10^ m^2^ s^−1^, respectively.^51^, ^52^, ^53^ The initial value of the 3D ReN culture MC was set to 1.65 × 10^−7^ mol m^−3^ considering Aβ production before connecting two chambers. The rate of Aβ production by AD cells was assumed to be 5 × 10^−12^ m per hour and diffusion through cells was assumed to be 2 × 10^−15^ m^2^ s^−1^ based on the detected Aβ amount from media in ReN cell media and bEC barrier MC.

*BACE1 Inhibitor Treatment*: After seeding and differentiating ReN‐AD cells for 2 d, 1 × 10^−6^ m of BACE1 inhibitor (LY2886721, Selleckchem, Houston, TX) was added with differentiation medium in the ReN media MC. Media was refreshed every day with 1 × 10^−6^ m of BACE1 inhibitor.

*ELISA Analysis*: The measurement of Aβ40 and Aβ42 was performed using the Meso Scale Discovery (MSD, Rockville, MD) 96‐well plate V‐PLEX Aβ peptide kits, as outlined in the manufacturer's protocol. The levels of IFNγ, IL‐1β, IL‐6, IL‐8, and TNFα were measured by MSD 96‐well plate Human Pro‐Inflammatory V‐PLEX Human Pro‐Inflammatory Assay kits. MMP‐2, MMP‐3, and MMP‐9 were measured by MSD 96‐well plate Human MMP ultrasensitive kits.

## Conflict of Interest

The authors declare no conflict of interest.

## Supporting information

Supplementary
