# Supplementary material for: Blood–Brain Barrier Dysfunction in a 3D In Vitro Model of Alzheimer's Disease
Source: Adv Sci (Weinh). 2019 Aug 12;6(20):1900962. doi: 10.1002/advs.201900962 (PMC6794630; doi:10.1002/advs.201900962)
Supplement: Supplementary file 1 — Supplementary [file ADVS-6-1900962-s001.pdf]

Copyright WILEY-VCH Verlag GmbH & Co. KGaA, 69469 Weinheim, Germany, 2019.

Supporting Information

for *Adv. Sci.,* DOI: 10.1002/advs.201900962

Blood–Brain Barrier Dysfunction in a 3D In Vitro Model of Alzheimer's Disease

Yoojin Shin, Se Hoon Choi, Eunhee Kim, Enjana Bylykbashi, Jeong Ah Kim, Seok Chung, Doo Yeon Kim, Roger D. Kamm, and Rudolph E. Tanzi**

Supporting Information

Blood Brain Barrier Dysfunction in a 3D in vitro Model of Alzheimer's Disease

Yoojin. Shin†, Se Hoon Choi†, Eunhee Kim, Enjana Bylykbashi, Jeong Ah Kim, Seok Chung, Doo Yeon Kim, Roger D. Kamm, Rudolph E. Tanzi*,*

Supplementary Materials

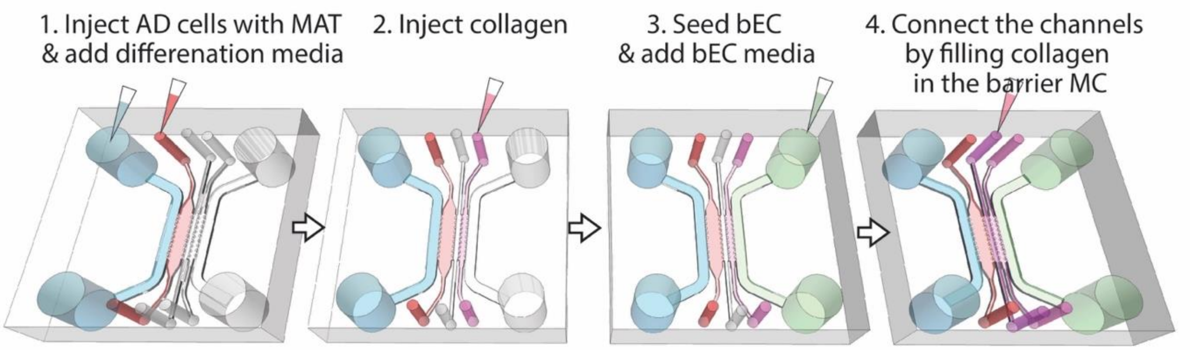

Supplementary Figure 1. The experimental procedures of developing a 3D WT/AD-BBB model.

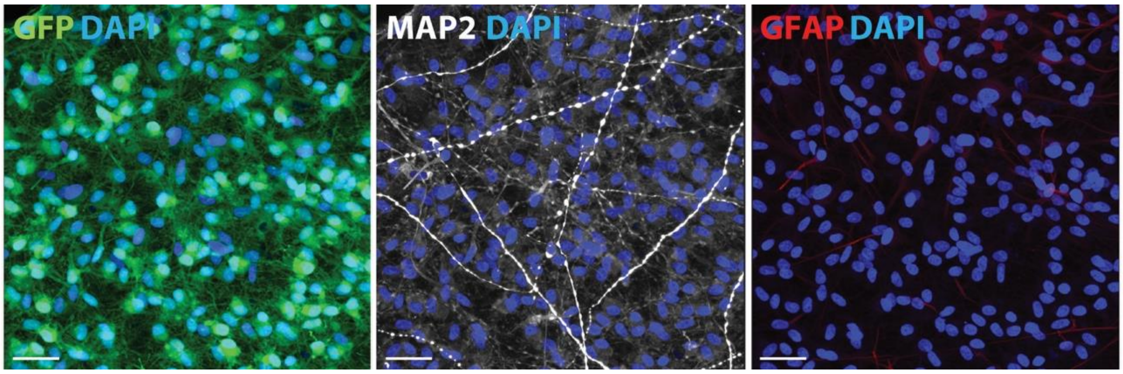

Supplementary Figure 2. Representative images of 2 weeks differentiated GFP expressing neural cells that are co-labeled with MAP2(white) and GFAP (red), and with DAPI staining for cell nuclei. Scale bars: 40 µm.

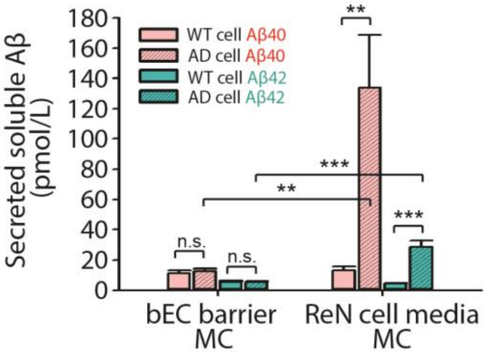

Supplementary Figure 3. The levels of soluble Aβ40 and Aβ42 in the bEC barrier MC and the ReN cell media MC in the 3D WT/AD-BBB model (n=4-8., **$p<0.01$ and ***$p<0.001$). Data are mean ± S.E.M. Statistical analysis was by †Student's t test.

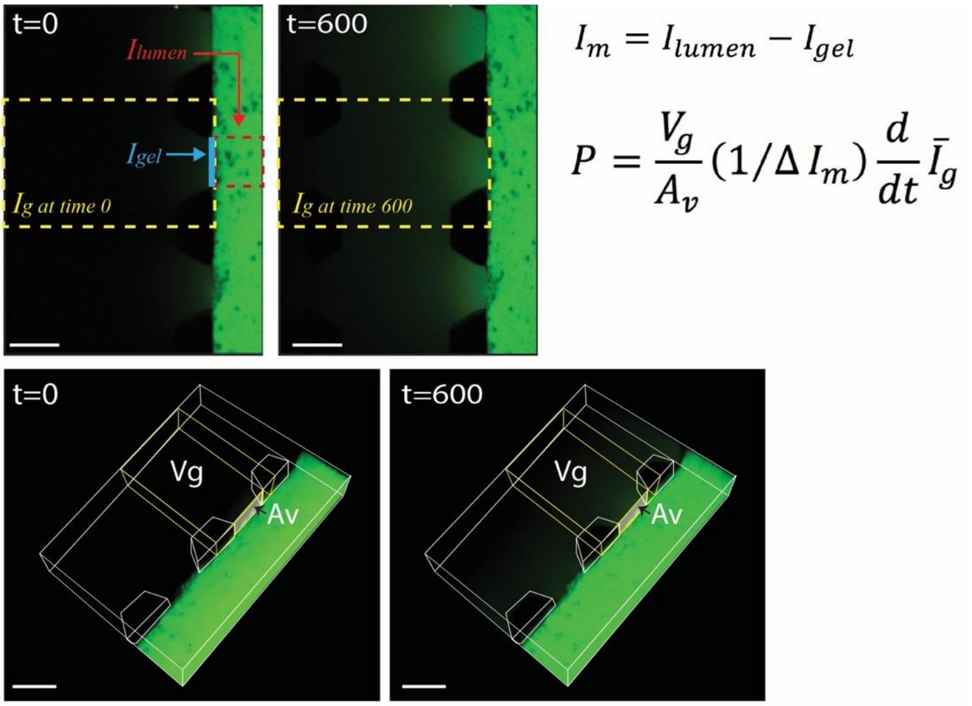

Supplementary Figure 4. Permeability values of bEC barrier in the microfluidic system were measured by introducing bEC medium supplemented with 10 μM dextran (40 kDa (FITC dextran) and 3kDa (Texas red)) into the BBB channel and monitoring FITC fluorescence every 5 min after the flow stabilization. The images were recorded at time 0s (top) and time 600s (bottom) after injection of 40kda dextran. The permeability was calculated by measuring the mean fluorescence intensity in a control volume (CV) defined in the collagen gel at time 0 and time 600s. Vg= gel volume in CV, Av=vessel surface area in CV, Ig=fluorescence intensity in gel, Im=fluorescence intensity in monolayer, (dI/dt)0: the rate of increase in intensity as dextran diffuses out of the BBB channel into the gel. Scale bars: 200 µm.

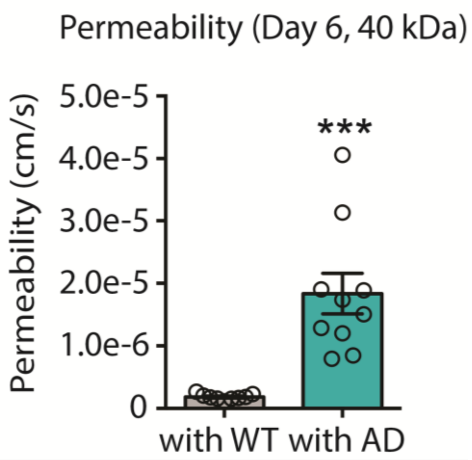

Supplementary Figure 5. The graph of permeability values of the bEC barrier in the 3D WT-BBB model and 3D AD-BBB model at co-culture day 6. (40 kDa, ***p<0.001) Data are mean ± S.E.M. Statistical analysis was by †Student's t test.

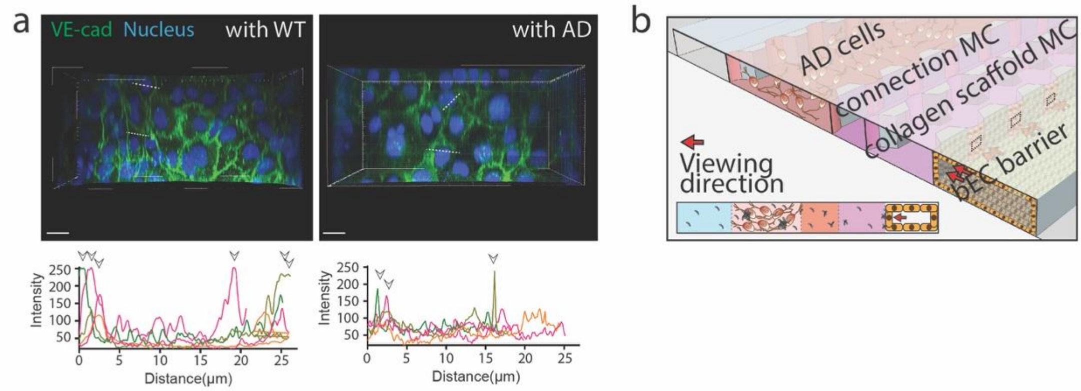

Supplementary Figure 6. a, Comparison of adherens junction protein expressions in the bEC barrier cultured with WT and AD cells in the 3D WT/AD-BBB model. The adherens junction protein expressions of VE-cadherin was visualized by immunofluorescence staining with an antibody against VE-cadherin with DAPI staining for cell nuclei. The graphs show that the intensity profile of VE-cadherin at the cell junctions. Arrows indicate VE-cadherin expression at the junctions of cells. Each colored line indicates the intensity profile of VE-cadherin expressed by randomly selected cells. Scale bars: 20 μm **b,** Schematic depiction of viewing direction for the images.

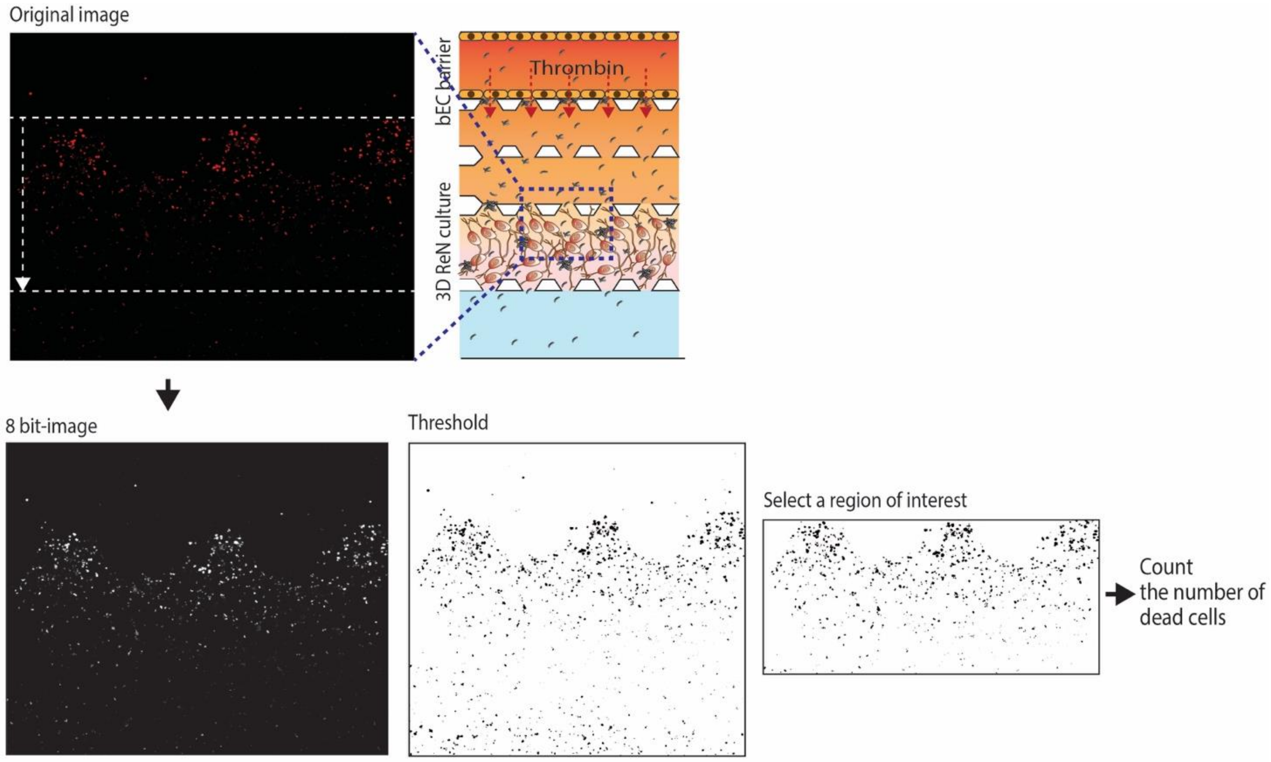

Supplementary Figure 7. Quantification method of cell death. The dead cells were visualized with the membrane-impermeant dye ethidium homodimer-1 (Red). Images were obtained from the confocal microscope.
